# Arabic Perspectives on Classification of Psychotic Disorders: A Historical Overview and Comparison With Contemporary Classifications

**DOI:** 10.31083/AP43494

**Published:** 2025-06-10

**Authors:** Homayun Shahpesandy

**Affiliations:** ^1^Great Oaks Inpatient Services, Doncaster and South Humber NHS Foundation Trust, DN4 8QN Rotherham, UK

**Keywords:** Arabic-Islamic psychiatry, psychoses, Tabari, Avicenna, Rhazes

## Abstract

This paper explores the perspectives of Arabic-scribing medical schools on the classification of psychotic disorders of the Middle Ages, the so-called “Islamic Golden Age”. Through an in-depth analysis of seminal texts in Arabic medical literature, including works by renowned scholars such as Tabari, Razi, Avicenna, and others, this paper elucidates the historical development of psychiatric taxonomy in the Arabic medical tradition. By examining the representations of “junun” (“madness”) or psychoses in these texts and comparing them with earlier European and current classifications, we aim to highlight the unique contributions of Arabic-scribing scholars to the field of clinical psychiatry. The Arabic taxonomy divides “junun” (“insanity”) into three major categories of (1) permanent insanities (“al-junun al-thābet”), (2) symptomatic illnesses (“a’rāz tābea-tul amrāz”), and (3) reactive. Arabic medical schools consider “insanity” as a primary brain pathology albeit with multifactorial etiology—a concept formulated by early Greco-Roman medicine, developed by Muslim physicians, and re-invented by Griesinger in the 19th century—known as the “organic model” of mental illnesses.

## Main Points

1. **Arabic Classification of Psychosis**: The Arabic classification of psychosis, notably under the term “junun,” categorized psychotic disorders into three subtypes: symptomatic, permanent, and reactive, reflecting a nuanced understanding of mental illness.
2. **Organic Model**: Arabic medical schools, influenced by Greco-Roman medicine, developed the “organic model” of mental illness, proposing that mental disorders arise from brain abnormalities—a concept later reintroduced by Wilhelm Griesinger in the 19th century.
3. **Cultural and Intellectual Exchange**: Translations of Arabic medical texts including those by Rāzi, Majusi, and Avicenna into Latin by figures like Constantinus Africanus and Gerardo de Cremona facilitated the transfer of Arabic medical knowledge to Europe, influencing Western medical practices.
4. **Long-lasting Influence**: The Arabic classification system remained in use in the Islamic world for centuries, influencing Persian and Turkish medical traditions before being gradually replaced by Latin-based nomenclature in the modern era.
5. **Legacy in Modern Psychiatry**: Arabic contributions to the understanding and classification of psychosis helped to shape contemporary psychiatric knowledge, emphasizing the importance of cultural diversity in the development of medical concepts.

## 1. Introduction

The term “mania” stems from two Proto-Indo-European words, “mnyo” and “men” meaning “to think”, “to remember”, and “desire”. The Greek word μ$\overset{´}{\eta }$νις (minis) translating as wrath, and μν$\overset{´}{\eta }$μη (mnimi) meaning memory, stem from the above roots. The term μαν$\overset{´}{\iota }$α (mania) refers to persistent rage and unrelenting desire, and the verb μα$\overset{´}{\iota }$νoμαι (mainomai) “to be mad”, or “to be furious”. The Greek μαν$\overset{´}{\iota }$α (mania) was translated into Latin as “insania” [1] a compound word consisting of “in” (not) and “sanus” (health) meaning “unhealthy” or “irrational”. Insania was translated into English as insanity or madness. Other, Latin-based translations of mania include vesania (insane), “ve” (not) and “sanus” (sane) [2], folia, folie (foolishness or lack of good sense) fury [1], and délire, used mainly in French psychiatric literature [3].

The Scottish physician William Cullen (1710–1790) [4], reformulated “insanity” by calling it “neuroses” (nervous disease), dividing it into comata, adynamiae, pasmae, and vesaniae (insanity). Mania and melancholia fell into vesaniae, which Cullen defined as disorders of judgement [4] or an “unusual and commonly hurried association of ideas” leading to a “false judgement” and generating “disproportionate emotions” [5].

Cullen’s concept of “neurosis” was substituted by the term “psychosis” or “intelligence neurosis”, by the German physician Carl Fridrich Canstatt (1807–1850) in 1841 [6, 7]. In 1845 the Austrian physician Ernest von Feuchtersleben (1806–1849) used the term “psychosis” as a synonym for psychopathy, emphasising both the change in the entire personality and the interaction between physical and mental processes [8, 9]. In the 2nd half of the 19th century, the term psychosis was used as a synonym for mental disorder, mental illness, and insanity [6]. Paul Möbius (1853–1907) in 1875 expanded the concept of psychosis distinguishing between endogenous psychoses (covering the spectrum of hysteria, melancholy, mania, and paranoia), and exogenous psychoses to illustrate the causation of mental disease through any extraneous influence, whether somatic or psychic [10]. Successively, Emil Kraepelin (1856–1926) and Eugen Bleuler (1857–1939) divided endogenous psychoses into manic-depressive and schizophrenia-spectrum disorders [11].

Further development of the Cullen’s concept of neurosis occurred at the end of the 19th century. The French Pierre Janet (1859–1947) [12] distinguished two categories of neuroses: hysteria and psychasthenia. Sigmund Freud (1856–1939) in 1898 divided neuroses into actual neuroses (further subdivided into anxiety neurosis, neurasthenia and hypochondria), where aetiology is not sought in infantile conflicts but in the somatic dysfunction of sexuality, and psychoneuroses, where the determining aetiological factor is psychic conflict and a compromise between desire and defence. The group of psychoneuroses included hysteria and psychoses, also labelled as “defensive psychoses”, or paranoia [12]. In 1924, Freud used the term “narcissistic neurosis”  to refer to psychotic illnesses such as dementia praecox (schizophrenia), paranoia, melancholia [6]. In the 19th century, the term neurosis included a whole range of affections, which can be characterised as follows: (a) their exact location in the body is known (cardiac neurosis, gastric neurosis) or their place is assumed, as in the case of hysteria (uterus) and hypochondria (alimentary canal); (b) they are functional affections, that means “without inflammation or damage to the structure” of the given organ, and (c) are considered diseases of the nervous system [13].

Nowadays, the term psychosis in both the International Classification of Diseases (ICD) and Diagnostic and Statistical Manual (DSM) refers to a collection of symptoms, such as delusions and hallucinations, disorganised thinking, speech and behaviours, accompanied with social dysfunction and loss of contact with reality [14, 15]. Despite its widespread adoption, the term “psychosis” lacks a unified definition in Romance languages, posing challenges for cross-cultural understanding and translation [9].

The Arabic term “junun” (“madness”) stems from the word “jinn” meaning “covered”, “hidden”, “unknown”, “idiopathic”, as well as “demon”, “fairy”, or “goblin” [16, 17]. Besides, the term “junun” has several other meanings, including “foolishness”, “going crazy”, “darkness of the night”, “the beginning of the night”, “burying the dead”, “covering up”, “bringing the earth’s plants to bloom”, “a fly buzzing”, “temptation”, and “passion”. The noun “majnun” means “mad” or “insane” [16]. The word mania and psychosis are translated into Arabic as “al-hawwas”; for example, “acute hallucinatory mania” (psychosis) is translated as “hawwas halwasi-yun hád”, “delirious mania” as “hawwas hadayāni”, “epileptic mania” (post-ictal psychosis) as “hawwas sar’ee” or alcoholic psychosis as “hawwas kuhulí” [18].

## 2. Objectives

This paper seeks to scrutinise the depictions of “junun” (psychoses) in Arabic medical texts of the Middle Ages and juxtapose them with earlier European, and contemporary perspectives, thereby enhancing our comprehension of the historical trajectory of psychiatry.

## 3. Methods

A rigorous literature search was conducted to explore the representations of psychotic disorders in Arabic medical literature. The following seminal texts were meticulously examined:

1. Firdaus-al-Hikmah (“The Paradise of Wisdom”) by of Abu-l Hasan Rabban Tabari (c.808–861). Tabari, born in Marv (present-day Turkmenistan), primarily resided in Baghdad throughout his life, serving as a court physician to Abbasid caliphs. He was a multifaceted scholar, philosopher, astronomer, linguist, and physician. Tabari authored numerous other books, including Kitab-ul Din-ul Dawla (The Book of Religion and Governance), “Kitab-ul Hifz-ul Siha” (The Book of Sustenance of Health), and Kitab fil amsāl-ul a’dāb alal mazāhib-ul Fars, wal Rum wal Arab (The Customs of Persians, Greeks, and Arabs). Notably, the Firdaus ul-Hikmah (The Paradise of Wisdom), the inaugural medical textbook in Arabic, was dedicated to the caliph al-Mutawakkil [16].
2. Kitab al-Hāwī fī-tibb (“The System of Medicine”) by Abu Bakr Mohammad Zakariya Râzī (c.865–925), known as Rhazes. Râzī, a celebrated Islamic physician and philosopher. Born in Ray (Iran), he initially pursued a career as a goldsmith. It is said that he commenced his medical studies at 40 and was a student of the renowned physician Tabari. He authored over one hundred works on metaphysics and medicine, including Al Faraj ba’da’sh-Shadda (Joy after Sorrow), Kitab fi al-Jadari wa al-Hasbah (Book on Measles and Smallpox), and Al Hāwī. In addition to his medical pursuits, Râzī dedicated his attention to Alchemy, on which he wrote twelve books. Râzī’s expertise in medicine earned him the title of “Galen of the Arabs” [16].
3. Kitābu’l Maliki (“The Royal Book”) by Ali Abbās al Majūsī (949–994), known as Hally Abbas. Born in Ahvaz (Iran), Majūsī was a pioneering physician and surgeon who made significant contributions to the medical field through his monumental work, Kāmil ul-Sinā’at’ ul-Tibya (The Comprehensive Book of Medical Art). This comprehensive medical encyclopaedia, also known as Kitābu’l Malikī (The Royal Book), dedicated to the Buyid king Adud-al-Dawla Fannā Khusraw, who ruled Iraq and southern Persia between 949 and 983, encompassed a wide range of scientific knowledge. Notably, the Malikī book served as the primary medical text utilised by physicians before Avicenna’s Qānūn; however, when comparing Qānūn and the Royal Book, Qānūn was deemed superior in terms of scientific theoretical knowledge, while the Royal Book exhibited superiority in practical application [16].
4. Al-Qanun fī al-Tibb (“Canon of Medicine”) by Abu Ali Husein ibn Abdullah ibn Sina, known as Avicenna (980–1037). Avicenna was born into a noble family in Balkh (Afghanistan). He is broadly regarded as one of the most prominent polymaths of the Islamic world. Avicenna is widely recognised as “the Philosopher of the East, the Proof of God unto His creature”. He authored an extensive body of work, comprising 450 books, including Kitab al-shifa (The Book of Healing), Kitab al-Najat (The Book of Deliverance), and the seminal medical text Qānūn fit Tib (“Canon of Medicine”) [16].

These texts were selected based on their historical significance and scholarly reputation within Arabic and Islamic medical traditions. The analysis focused on identifying and comparing the classifications and conceptualisations of psychotic disorders as presented in these foundational works.

## 4. Results

The first medical textbook in Arabic, “Firdaus as-Hikmah” (The Paradise of Wisdom) by Tabari was composed in the year 848 A.D. Tabari, under the “Head illnesses” classifies neuropsychiatric disorders into thirteen syndromes such as:

Al-sar’a (epilepsy), also called “al-amrāz al-kāhení” (“soothsaying illness”),

Al-waswassah (obsessive disorder),

Al-hazyān (delirium),

Fasâd al-takhayyol (impaired imagination),

Fasâd al-aqhl (thought disorder),

Al-nasyān (dementia),

Al tawahush fi al-barari ma’a wahsh (“isolation in the wilderness with animals”),

Al-sahar (insomnia),

Al-sobāt (hypersomnia),

Al-dawí (tinnitus),

Al-dawwār (vertigo),

Al-warram (inflammation) and

Al-suda’a (cephalalgia), Table 1.

**Table 1.** **Tabari’s classification of “Brain Diseases”**.

Tabari’s classification		Contemporary equivalent	
1. Al-sar’a, “efilebsiya” or “al-amrāz al-kāhení” (“soothsaying illness”)		1. Epilepsy	
2. Al-suda’a		2. Cephalalgia	
	a. *Al-sanurta*		a. *Al-sanurta *(defined as the most severe form of headache, which affects the whole head)
	b. *Al-shaqīqha*		b. Migraine
3. *Al-dawí (al-tannin)*		3. Tinnitus	
4. *Al-dawwār*		4. Vertigo	
5. *Al-warram*		5. Inflammatory diseases	
6. *Al-nasyān*		6. Dementia	
7. *Al-hazyān*		7. Delirium	
8. *Fasâd al-takhayyol*		8. Impaired imagination	
9. *Fasâd al-aqhl*		9. Thought disorder	
10. *Al tawahush fi al-barari ma’a wahsh*		10. “Isolation in the wilderness with animal”	
11. *Al-sahar*		11. Insomnia	
12. *Al-waswassah*		12. Obsessive disorder	
13. *Al-sobāt*		13. Hypersomnia	

Likewise, Tabari talks about “sobāt” (mutism), “humm-a al-hubbi” (“love fever”), and “humm-al-sehr” (“fever from enchantment”) [19, 20]. Tabari, hence establishes the first classification of mental disorders in Arabic and post-Islamic medicine.

The second and more influential classification of “al-junun” (“madness”) comes from Râzī in his “Al-Hāwī fil Tib” which divides insanities into two categories:

Al-junun al-thābet or “permanent madness” (permanent psychosis), and

A’rāz tābea-tul amrāz symptomatic or organic mental disorders [21, 22].

As depicted in Table 2, the classification of insanity portrayed by Razi was reformulated and expanded by Avicenna, and other physicians based on their aetiology, clinical presentations and course, into the following categories:

**Table 2.** **Razi’s classification of “Head Diseases”**.

Razi’s classification		Contemporary equivalent	
*Al-sakta*		Stroke	
*Al-falaj*		Paralysis (including monoplegia, hemiplegia and paraplegia)	
*Al-khadr*		Numbness (including hypesthesia and paraesthesia)	
*Al-ra’asha*		Tremor	
*Usr-ul-hisi*		Dysesthesia	
*Al-ekhtelāj*		Convulsion disorders	
*Al-dawwār*		Vertigo	
*Al-dawí*		Tinnitus	
*Al-sidr*		Syncope	
*Al-laqhawa*		Facial nerve palsy (Bell’s palsy)	
*Al-tashannoj*		Spastic disorders	
*Al-tamuddud*		Dilatation and stretching	
*Al-kazāz*		Tetanus	
*Al-sar’a*		Epilepsy	
*Um-ul-sibyān*		“The mother of children” (infantile seizures)	
*Al-kābus*		Nightmares	
*Al-faz’a fin-nawm*		Nocturnal panic attacks or night terror	
*Lethargus*		Lethargus (lethargic encephalitis)	
*Phrenitis*		Phrenitis (meningitis and/or encephalitis)	
*Al-sahar*		Insomnias	
*Awrām hārat fir-rā’s*		“Hot tumours” (inflammatory diseases) of head	
*Al-suda’a*		Cephalalgia	
	■ *Al-shaqīqha*		■ *Trigeminal neuralgia*
	■ *Bayda*		■ *Migraine and hemicrania*
	■ *Al-taza’zu damāgha min darba*		■ *Traumatic and shaken brain syndrome*
	■ *Al-mā fir-rā’s*		■ *“Water in the head” - hydrocephalus*
*Al-malīkholyâ*		Melancholia	
*A’rāz tābea-tul amrāz:*		Symptomatic insanities:	
	■ *Ekhtelāt-ul-takhayyol*		∙ *Corruption of perception*
	■ *Ekhtelāt-ul-fekr*		∙ *Corruption of thought*
	■ *Ekhtelāt-ul-aqhl*		∙ *Corruption of the mind*
	■ *Phrenitis-induced insanity*		∙ *Psychosis due to phrenitis or organic psychosis*
	■ *Al-hazyān*		∙ *Organic delirium*
	■ *Ekhtelāt al-aqhl al-kayin-al-sharāb*		∙ *Corruption of the mind due to alcohol - alcoholic psychosis*
*Al-junun al-thābet:*		Permanent insanities:	
	∙ *Al-junun-al hāyej*		∙ *Ferocious madness - mania*
	∙ *Qhutrub*		∙ *“Dragonfly” - agitated mania*

Symptomatic insanities “a’rāz tābea-tul amrāz”

Permanent insanities, “al-junun al-thābet”, and

Reactive insanities, Table 3 (Ref. [3, 23, 24, 25, 26, 27, 28, 29, 30]).

**Table 3.** **Comparison of the Arabic classification of psychotic disorders vs. earlier European classification and the 10th revision of the International Classification of Diseases (ICD-10)**.

Arabic classification		ICD-10
1. *A’rāz tābea-tul amrāz* (symptomatic psychotic disorders)		F06 Mental and behavioural disorders due to brain damage, dysfunction or physical illness:
	1.1. *Ekhtelāt-ul-takhayyol* (corruption of imagination)	F06.0 Organic hallucinosis
	1.2. *Ekhtelāt-ul-fekr* (corruption of thoughts)	F06.2 Organic delusional disorder
	1.3. *Ekhtelāt-ul-aqhl* (corruption of the mind) or *junun* (insanity)	F06.2 Organic delusional [schizophrenia-like] disorder
	1.4. *Sobārā* (psychosis due to direct or indirect brain damage)	F06 Mental disorders due to brain damage and dysfunction and to physical disease
		F07.1 Postencephalitic syndrome disorder
		F09 Unspecified organic or symptomatic mental disorder
	1.5. *Sokāt* (organic mutism)	F06.9 Unspecified mental disorder due to brain damage and dysfunction and to physical disease
		F09 Unspecified organic or symptomatic mental disorder
	1.6. *Tashwish* (memory illusion)	F06.8 Other specified mental disorders due to brain damage and dysfunction and to physical disease
	1.7. “Rotating noises in the brain”	F06.0 Organic hallucinosis
		“Syrigmus” described by Sauvages in 1763 as “unreal sounds perceived in the air” [3]
	1.8. *Ekhtelāt al-aqhl al-kayin-al-sharāb* (alcohol-induced insanity)	F10.7, ICD-10 Residual, and late-onset psychotic disorder due to alcohol
2. *Al-junun al-thābet *(permanent psychotic disorders)		F20 - F29 Schizophrenia, schizotypal and delusional disorders:
	2.1. *Al tawahush fi al-barari ma’a wahsh (“isolation in the wilderness with animals”)*	F20.6 Simple schizophrenia
	2.2. Mania	F20 Schizophrenia-spectrum disorders
	2.3. *Qhutrub* (dragonfly) - agitated insanity	F20.9 Unspecified schizophrenia
		Lycanthropy described by Bayfield in 1663 corresponding with the description of “qhutrub” [23]
	2.4. *Da’al-kalb* (mixed psychosis)	F23.8 Acute and transient psychotic disorder, F23
		F25.2 schizoaffective disorder, mixed type
		F31.6 Bipolar affective disorder, current episode mixed
		“Dysthymia mutabilis” described in 1844 by Fleming [24]
		“La folie à double forme”, introduced in 1854 by Baillarger and “folie circulaire” by Falret [25]
		“Folie intermittentes” described in 1893 by Magnan [26]
		Manic-depressive insanity introduced in 1899 by Kraepelin [27]
		Bipolar affective disorder portrayed by Angst and Perris in 1966 [25]
	2.5. *Junun al-dawrí* (cycloid insanity) and *junun -ul mutbeqh* (“absolute” insanity)	F31 Bipolar affective disorder
		“La manie périodique ou intermittente” depicted by Pinel in 1798 [30]
		“Cycloid psychoses” defined by Kleist in 1928 [28, 29]
3. Reactive psychotic disorders		
	3.1. *Al-humma min-al-sehr* (“Fever due to enchantment”)	F23.3 Psychogenic paranoid psychosis
	3.2*. Haymān* or *eshqh* (“love madness”)	F32.8 Atypical or “masked” depression
		F45.0 Somatisation disorder

### 4.1 “A’rāz tābea-tul amrāz” (Symptomatic Psychotic Disorders)

Symptomatic mental disorders are considered as conditions resulting from primary, internal factors such as infections or external factors like heatstroke or a blow to the head) or secondary damage or dysfunction of the brain, by illnesses of other organs, including “sarsām” (inflammation of head — encephalitis, meningitis) “barsām” (inflammation of the chest — pneumonia, pleurisy) urine tract infections (UTI), etc. [20, 21, 22, 31].

### 4.2 “Ekhtelāt-ul-takhayyol” (the Corruption of Imagination)

The “corruption” of imagination is portrayed as a syndrome when an individual’s perception (“al-his”) is impaired, whilst their thinking, (“al-fahm”) is intact, characterised by illusions, simple and complex hallucinations, such as “seeing people, fire, water, etc, or being observed picking up chips and hay, hearing sounds, or sensing smells that have no external reality” [20, 31]. Tabari believes if the frontal parts of the brain (“centrum of fantasy”) are damaged, patients experience complex visual hallucinations “like the man who was shouting at a group of people playing music opposite of his house” [19, 20]. Avicenna associated this syndrome with the damage of the “middle brain” [31]. The syndrome of impaired imagination matches with the organic hallucinosis, ICD-10 F06.0 [14].

### 4.3 “Ekhtelāt-ul-fekr” (the Corruption of Thought)

The “corruption of thought” is outlined as a condition, when the perception is intact and “patients perceive the outside reality as it is”, nonetheless, their thinking is impaired, “like a man who closed the door behind himself, opened the windows and threw everything he could find in the room” [19, 20] or “like the man who throws a carpet and dishes from the roof (of his house) at a woman in the garden of the house. This person’s perception is intact because he recognises the objects (carpet and dishes), but he does not understand that he should not throw those objects (at the woman) in the garden” [21, 22]. This syndrome is linked with damage to the central parts of the brain, ‘the place of thinking’ [19, 20], and pathologies of the frontal lobe [31].

### 4.4 “Ekhtelāt-ul-aqhl” (the Corruption of the Mind), or Junun (“Madness”) 

This condition is described as a syndrome when there is a profound change in patients thinking, emotions and behaviours, compared to their pre-morbid personality; “they say things they normally not say, like things they (normally) should not like, wish unreasonable things, desire for undesirable, do things they should not do, or hate things that they normally, do not hate” [31]. Tabari associates this illness with the damage of the posterior cerebellar areas (“centrum of memory”) caused by their “heating” (inflammation) and “dryness” (dehydration). The affected individuals exhibit complex hallucinatory experiences, thought disorders including delusions; “some believe they are made of ceramic and are scared something will hit them and they will break”, some believe they are “camels and run away from human”, or imagine they were “transformed to rooster and make noises like roosters” [19, 20]. Avicenna relates this syndrome with the impairment of central parts of the brain [31].

Both the above-mentioned syndromes resemble the organic delusional disorder, ICD-10 F06.2, characterised by predominant delusional beliefs accompanied by hallucinations [14].

### 4.5 “Sobārā” (Organic Psychosis)

“Sobārā” is portrayed as an organic “madness” due to either primary brain damage, such as “sarsām” or phrenitis (meningitis or/and encephalitis) or a secondary brain dysfunction because of “barsām” (pneumonia and pleurites), UTI and other infections, characterised by agitation, confusion, cognitive impairment and psychotic phenomena [21, 22, 31].

“Phrenitis” in ancient Greece refers to acute inflammation of the mind and body [32], or describes any mental disorder associated principally with fever, but also cranial traumatism and other somatic causes [33]. Phrenitis alongside “lethargus” used in the mediaeval Islamic medicine as “qeranits” and “lethargus” denote agitated and inhibited delirium [34]. Celsus who introduced the term “delirium” into medicine gave a detailed description of phrenitis, considering it as equivalent to mania (“madness”) and dividing it into acute phrenitis, which he called delirium, characterised by febrile paroxysms, incoherent speech and confusion, and chronic insanity or “phrensis” manifested by persistent dementia; “when the patient is so far in his senses, he entertains himself with futile imaginations; and the mind is at the mercy of these imaginations” [35].

“Sobārā” aligns with disorders ordered in the ICD-10, under the category, F06, Other mental disorders due to brain damage and dysfunction and physical diseases [14], and F07.1 Postencephalitic syndrome, presenting with nonspecific symptoms and varying from individual to individual and from infectious agents [14].

### 4.6 “Ekhtelāt al-aqhl al-kayin-al-sharāb” (Alcohol-induced “Madness”)

Alcohol in moderation is recommended for its “antidepressant” and anxiolytic effects; however, excessive consumption and frequent intoxication are not recommended, due to the toxic effects of alcohol on the brain and liver [31]. Alcohol-induced “insanity” is considered a benign form of psychosis with a good prognosis, characterised by delusional beliefs, hallucinations, anger, hostility [21, 22] and personality changes, such as “ignoring religious and social issues”, accompanied by the decline of cognitive functions, perception, comprehension and understanding [36, 37]. Alcohol-induced “madness” as depicted here, resembles the F10.7, ICD-10 Residual, and late-onset psychotic disorder due to alcohol, characterised by changes in cognition, affects, personality or behaviours [14].

### 4.7 “Sokāt” (Organic Mutism)

Labelled as a condition developing from the excessive heat or aridity of “both sides” (temporal lobes) of the brain [19, 20]. The syndrome of organic mutism relates to other specified mental disorders due to brain damage and dysfunction and to physical disease, F06.8, ICD-10 [14].

### 4.8 “Tashwish” (Memory Illusion)

Coupled with the damage of the frontal brain lobe and described both as an olfactory hallucinatory syndrome “when an individual senses smells without its external reality” and a memory illusion “when the mind is disordered, and the person remembers events that he or she has never seen or heard of” [31]. The syndrome of “tashwish” because of its ambiguity, could be classified as an Unspecified mental disorder due to brain damage and dysfunction, F06.9, ICD-10 [14].

### 4.9 “Rotating Noises in the Brain”

A rare hallucinatory syndrome presenting with elementary auditory hallucinatory experiences in the form of sounds accompanied by insomnia and anxiety due to “vapours getting stuck and circling in the brain” [20]. The French physician, Boissier de Sauvages (1706–1767) portrayed a similar syndrome, “syrigmus” described as “unreal sounds perceived in the air” in 1763 [3]. The syndrome of “rotating noises in the brain” resembles Organic hallucinosis, F06.0, a hallucinatory disorder occurring in clear consciousness that may or may not be recognised by the person as such [14].

### 4.10 Al-junun-al-thābet (Permanent Insanities)

Permanent “insanities” are supposed to be “persistent disturbances of the mind” [21, 22] of multifactorial aetiology, where “physical” or biological (temperament, humours, physical predisposition, personality) “non-physical” or psychological (excessive emotions as sadness, fear, anxiety) and environmental factors interplay in their aetiology [38, 39].

### 4.11 “Al tawahush fi al-barari ma’a wahsh” (“Isolation in the Wilderness With Animals”)

Tabari’s avoidance of the term “insanity” in favour of “isolation in the wilderness with animals” offers a unique perspective on psychosis. This term encapsulates a spectrum of symptoms, ranging from anxiety and agitation to disorganized behaviours and cognitive deficits. Individuals experiencing this state may exhibit obsessive symptoms, forgetfulness, confusion, and hostility. Interestingly, Tabari notes a propensity for affected individuals to abandon human company and seek solace among savage animals. Moreover, hallucinations and nihilistic delusions, such as the belief that the sky will fall or delusions of metamorphosis such as being transformed into animals, are highlighted as prominent features. Tabari attributes the origins of this illness to environmental factors such as extreme heat or dryness (caused either by external factors such as sun heat, or infections and other febrile states), drawing parallels to the desiccating effects of fever or pleurisy [19, 20]. A similar clinical picture is described by Aretaeus, in people “who suffer from melancholia are suspicious of poisoning, or flee to the desert from misanthropy, or contract a hatred of life. They complain of life and desire to die; they become ignorant of all things or forgetful of themselves and live the lives of the inferior animals” [40]. The syndrome “isolation in the wilderness with animals” is possibly the first description of schizophrenia-spectrum disorder, namely simple schizophrenia in Arabic medicine, corresponding to F20.6, characterised by significant changes in personal behaviours, manifest as a marked loss of interest, idleness, and social withdrawal [14].

### 4.12 Mania (“madness”) 

There is no mention of mania in the early Arabic medical texts, instead, the term “ekhtelāt-al-aqhl” (“the corruption of the mind”) or “junun” (“madness”) is used. Razi describes mania as “al junun ul-hāyej” or “ferocious madness” with its characteristic symptoms of agitation, aggression, fear and paranoia, delusional thinking, and impaired judgment [21, 22].

Avicenna’s exploration of mania and melancholia sheds light on the complex interplay between bodily humours, in particular “accumulation of the black bile or burnt bile in the frontal parts of the brain or its substance” and their clinical manifestations. Avicenna distinguishes two variants of mania such as “sobārī” (agitated) and “hārī” (inhibited) mania each characterised by distinct features and behaviours, highlighting the heterogeneity of psychosis and the importance of individualised approaches to treatment [31].

Avicenna explains although mania and melancholia have the same pathophysiology, they differ in their clinical symptomatology; whilst in melancholia, pessimism, negative thinking, fear, and social isolation prevail; in mania, “the worst form of insanity”, paranoia, constant anxiety, agitation, vindictiveness, hostility, and ferocity are the characteristic symptoms [31].

Mania as described in Arabic medical literature has no relation with the current understanding of mania, as an affective disorder, but is understood as a severe form of psychosis, from the schizophrenia-spectrum disorders, F20, ICD-10 [14].

### 4.13 “Qhutrub” (dragonfly) — Agitated Psychosis

“Qhutrub” or dragonfly is described as a form of treatment-resistant “madness”, characterised by erratic, and disorganised behaviours, restlessness and agitation, “wandering from night to dawn”, especially in cemeteries. Seeking solace, they retreat to cemeteries and places associated with the deceased, reacting aggressively to any unexpected encounters. These individuals isolate themselves for days, avoiding social interactions and predominantly emerging at night. Their restless behaviours are marked by aimless wandering, seemingly detached from their surroundings. Despite their frenzied movements, they pose minimal harm to others. Individuals afflicted by “qhutrub” are described as having distinctive physical appearances such as gloomy countenance, yellowish complexion, deformed faces, stunned eyes, dry tongue, and insatiable thirst. Persistent ulcers on their legs and lacklustre eyes further characterise the condition [21, 22, 31, 38, 39]. Qhutrub is believed to be hereditary [38, 39] and occurs mostly in the second month of winter (Aquarius) [31].

The term “qhutrub” originates from an insect (dragonfly) that skims the water’s surface erratically, mirroring the patient’s perpetual motion and desire to escape. Other interpretations liken qhutrub to mythical creatures like a masculine giant or a hairless wolf [31].

The clinical picture of “qhutrub” resembles lycanthropy commonly described by medieval European physicians. The Italian physician Donatus ab Altomari’s (1506–1562) description of lycanthropy as “they have usually hollow eyes, scabbed legs and thighs, very dry and pale” [23] mirrors the description of “qhutrub”. English physician Robert Bayfield (1630–1690) in 1663 gives a depiction of lycanthropy, that remarkably resembles “qhutrub” in the Arabic medical literature. Bayfield wrote: “Lycanthropy, is a disease, in which man run barking and howling about graves and fields in the night, lying hid for the most part all day, believing they are wolves…”

Clinical presentation of “qhutrub” relates to schizophrenia-spectrum disorders, particularly, with Unspecified schizophrenia, F20.9, ICD-10 [14].

### 4.14 “Dâ-al-kalb” (“Dog’s Disease”) or “Mixed Psychosis” 

“Dā al-kalb” (“the dog’s disease”) is regarded as a subtype of mania (insanity), prevalent during the spring and summer with distinctive symptoms reminiscent of behaviours observed in dogs. “Dā al-kalb” is characterised by a mixture of anger and playfulness, alternating between periods of calmness and hostility. Moderate forms of “dā al-kalb” manifests with mild features of pessimism and paranoia [31], however, its severe forms called “junun al-sabo’í” or “savage madness”, manifest with severe agitation and hostility [16, 31].

The concept of “mixed insanity” corresponding to the clinical picture of the “dog’s disease” (da’al-kalb) has its roots in ancient Greek medicine but was not known to European physicians until the 19th century. In 1844 German physician Carl F. Fleming (1799–1880) in his classification of “dysthymia” (affective disorders) portrays “dysthymia mutabilis” or mixed affective disorder [24]. In 1854 French Jules Baillarger (1809–1890), described a variant of “insanity” he called “la folie à double forme”, and Pierre Falret (1794–1870) “folie circulaire” [25]. In 1893, V. Magnan (1835–1916) labelled “folie intermittentes” or “intermittent madness” [26]; all defined as a form of mixed insanity characterised by the existence of both manic and depressive episodes. Further evolution of this concept led to Kraepelin’s hypothesis of manic-depressive insanity, introduced in 1899 [27], and subsequently, re-named bipolar affective disorder by Angst and Perris in 1966 [25].

The da’-al-kalb clinical picture to some extent is akin to mixed episode of bipolar affective disorder, and schizoaffective disorder, mixed schizophrenic and affective psychosis, F25.2, ICD -10, or perhaps mixed episode of bipolar affective disorder, F31.6 characterised by alternation of manic, hypomanic and depressive symptoms [14].

### 4.15 “Junun al-dawrí” (Cycloid Insanity) and “Junun mutbeqh” (“Absolute” Insanity) 

“Junun edbāri” (cycloid insanity) is defined as a form of chronic insanity commonly exacerbating in spring but during the rest of the year is in remission [16]. It appears that “junun edbāri” is a synonym for seasonal melancholia described by Rhazes attributing its occurrence in spring to the stimulation of humours by the natural environment [21, 22]. Nonetheless, Avicenna believes “junun edbāri” is a variant of “mania” (“madness”) occurring in spring and early summer [31]. It is believed that Karl Kleist (1879–1960) who in 1928 first introduced the concept of “cycloid psychoses”, described them as “bipolar disorders, that do not belong to the category of manic-depressive insanity” [28, 29]. Evidence, however, suggests that it was Pinel who in 1798 introduced the term “la manie périodique ou intermittente” (periodic or intermittent mania), resembling “junun edbāri”, into modern psychiatry [30]. Pinel, considered periodic or intermittent insanity as a form of paroxysmal, relapsing “insanity”, characterised by a high degree of physical and mental energy, without delusion and independent of the influence of the seasons [41]. “Junun dawrí” relates to F31 Bipolar affective disorder [14].

In contrast to cyclic insanity (“junun edbāri”) “junun mutbeqh” (absolute insanity) or complete insanity (“junun al- mostau’ib”), is described as a form of “insanity that totally covers the mind”. What is more, “absolute” insanity is defined as “insanity” that persists for at least one month according to the Abu Hanifa (699–767A.D.), the founder of the Hanafi school of Islamic jurisprudence. Nonetheless, according to Abu Yusuf al-Kofi (732–801 A.D.) Abu Hanifa’s disciple and the chief justice during the reign of Harun ar-Rashīd (786–809) of the Abbasid caliphate (749–1258) the “absolute” insanity persists most of the year [16]. Interestingly, ICD-10 requirement for diagnosis of schizophrenia is one month or more, (for residual, and simple schizophrenia one year), and for persistent delusional disorder, at least three months [14].

## 5. Reactive Psychotic Disorders

### 5.1 “Al-humma min-al-sehr” (“Fever due to Enchantment”) 

Believed to be resulting from magic, manifested by symptoms of “loss of mind and thirst” [20]. The ancient phenomena of bewitchment, existing for millennia, and still observed in clinical practice, reflects socio-cultural beliefs, and not the opinions of the medical professionals. The syndrome of “fever due to enchantment” could be included under the Psychogenic paranoid psychosis, F23.3, ICD-10 [14].

### 5.2 “Haymān” or “Ishq” (“Love Madness”)

Portrayed as a psychogenic reactive psychotic state, manifesting with grief, worry, agitation, confusion, insomnia, restlessness, and fever [38, 39] as well as bewilderment of the mind, silence, weakness of sexual desire, shame and happiness when hearing the name of the beloved [20]. Avicenna paints love madness as an obsessive malady akin to melancholy, arising from the total immersion of one’s thoughts into fantasies and images of the beloved. Psychological symptoms include low mood, mood swings, poor appetite, and insomnia. Physiologically, the illness manifests in rapid eye movements, dry eyes, and sunken eye sockets, indicative of sleep deprivation. In terms of management, Avicenna advocates for a holistic approach. He recommends the administration of sedative medication and nutritious food. Psychologically, he suggests that the physician should counsel the patient that their thoughts are indicative of obsession and a form of madness. Additionally, Avicenna recommends inviting elderly women or hermaphrodites to gather around the patient and engage in conversations about their lover, attributing negative qualities to the individual to foster animosity towards the beloved. Alternatively, the physician can occupy the patient with various games or hunting activities. Furthermore, Avicenna suggests that having the patient sleep with slave girls can be an effective treatment for this condition [31].

Depending on its clinical manifestation; if the depressive symptomatology dominates the clinical presentation, “love madness” could be classified as atypical or “masked” depression, F32.8 [14]. Alternatively, the syndrome of “love madness” could be classified under somatisation disorder, F45.0 [14].

## 6. Conclusion

The classification of mental illness has undergone a significant evolution over the centuries, drawing upon contributions from diverse cultural and intellectual traditions. The Arabic classification of psychosis stands as a pivotal milestone in the history of psychiatry, illuminating the understanding of mental illness as perceived by Arabic and Islamic medical schools. This classification underscores the paramount importance of cultural diversity in shaping the conceptualisation of psychiatric knowledge.

Within the Arabic classification, the term “Junun” (psychosis) is categorised into three primary subcategories: (1) symptomatic, (2) permanent, and (3) reactive psychotic disorders. Notably, Arabic medical schools categorise “insanity” as a primary brain pathology, albeit with a multifactorial aetiology—a concept initially introduced by early Greco-Roman medicine, refined by Muslim physicians, and subsequently reintroduced by Wilhelm Griesinger (1817–1868) in the 19th century. This concept, known as the “organic model” of mental illnesses, posits that mental disorders arise from organic abnormalities in the brain.

The Arabic classification of psychotic disorders was a progressive process that established a common nomenclature within the Arabic-speaking and broader Islamic medical communities, facilitating mutual understanding among experts. Moreover, the developed taxonomy exhibited relative precision and stability, aligning with contemporary classification systems. Particularly, the Arabic taxonomy of psychosis had a profound influence on the development of medical nomenclature throughout Islamic civilisation, including Persian and Turkish medical terminology, two prominent medical traditions within the Islamic world. Furthermore, it is plausible that early European physicians were acquainted with the Latin-translated scripts of Razi, Majūsī, Avicenna, and other Arabic-writing physicians, leading to their influence. In the 11th century, Constantinus Africanus (approximately 1098) translated Arabic medical scripts, including Razi’s Al-Hāwī, Majūsī’s The Royal Book, and Avicenna’s Canon of Medicine, into Latin [42, 43]. Additionally, the renowned Italian translator Gerardo de Cremona (c. 1114–1187) translated over eighty Arabic scientific and medical books into Latin [44], which served as the primary scientific language in Europe until the 19th century. Similarly, Avicenna’s Canon was a prominent medical textbook in European universities until the 17th century [45].

The classification formulated by Râzi subsequently reformulated and expanded by Avicenna and other esteemed physicians of the era, remained in use within the Arabic and Islamic medical communities until the past century. However, it was eventually replaced by Latin-based vocabulary reflecting the international classification of diseases.
